# Sirt3 targets mPTP and prevents aging in the heart

**DOI:** 10.18632/aging.100266

**Published:** 2011-01-17

**Authors:** Junichi Sadoshima

**Affiliations:** Cardiovascular Research Institute, Department of Cell Biology and Molecular Medicine, UMDNJ, New Jersey Medical School, Newark, New Jersey, 07103, USA

The incidence of heart failure dramatically increases with aging. An important feature of cardiac aging is an increase in susceptibility to stress, such as ischemia and hemodynamic overload [1]. The heart is an organ in which mitochondria are abundant and mitochondrial dysfunction caused by oxidative stress is believed to be an important cause of aging and failure. Mitochondrial permeability transition pore (mPTP) opening, a hallmark of mitochondrial dysfunction, causes release of pro-apoptotic factors and depletion of ATP, and eventually induces apoptosis and necrosis of cardiomyocytes depending upon the severity of ATP depletion. Elucidating the molecular mechanism by which mPTP opening is regulated during aging would provide an important clue for developing modalities to reduce the incidence of heart failure in elderly patients.

In a recent publication, Hafner *et al.* showed that Sirt3 plays an essential role in fighting against cardiac aging and increases the tolerance of the heart against hemodynamic overload [2]. Sirt3 belongs to the sirtuin family proteins, which deacetylate cellular proteins in an NAD+-dependent manner. Sirt3 is localized primarily at mitochondria and deacetylates cyclophilin D, a key component of the mPTP, thereby inhibiting mPTP opening. This in turn reduces oxidative stress and eventually slows down cardiac aging.

Cardiac hypertrophy is often accompanied by oxidative stress, which causes oxidation and dysfunction of mitochondrial proteins, leading to further increases in mitochondrial oxidative stress through an amplification process called reactive oxygen species (ROS)-induced ROS release. Transition from hypertrophy to heart failure is almost always accompanied by increased oxidative stress and mitochondrial dysfunction, which trigger another vicious cycle consisting of oxidative stress, mitochondrial dysfunction and cardiac hypertrophy/dysfunction. This often makes it difficult to determine whether a given intervention acts primarily on cardiac hypertrophy, oxidative stress, or mitochondria to improve overall cardiac function, since all are affected concomitantly. In this regard, the fact that Sirt3 localized in mitochondria directly prevents mPTP opening by modulating cyclophilin D strongly suggests that Sirt3's primary action is to suppress mPTP opening and consequent mitochondrial dysfunction, and that the suppression of cardiac hypertrophy could be secondary to the suppression of mitochondrial/cardiac dysfunction [2]. The fact that Sirt3 inhibits cardiac hypertrophy was also demonstrated by Sundaresan *et al.*, where the authors proposed that Sirt3 deacetylates FoxO3 in the cytosol and induces nuclear translocation of FoxO3, which promotes transcription of anti-oxidants [3]. However, modulation of FoxO3 was demonstrated only in the presence of overexpression of Sirt3 and, thus, whether endogenous Sirt3 deacetylates FoxO3 in the cytosol remains to be shown. Whether or not Sirt3 has an additional direct effect upon cardiac hypertrophy independent of mPTP opening remains to be clarified.

Judging from the fundamental involvement of mPTP opening in myocardial cell death and the development of heart disease, modulation of Sirt3 could be an important modality for medical treatment of heart failure. Sinclair and colleagues have shown that the NAD+ biosynthetic enzyme Nampt plays an important role in maintaining NAD+ content in mitochondria, and that its protective effects are mediated through Sirt3 and Sirt4 [4]. It would be interesting to test whether interventions upon Nampt achieve the same effect in the heart as those upon Sirt3. We have shown recently that cardiac specific overexpression of Nampt induces protection against myocardial ischemia, in part through activation of autophagy, another cellular mechanism implicated in aging and lifespan extension [5].

It remains to be shown how the activity of endogenous Sirt3 is regulated by aging in the heart. If the activity of Sirt3 goes down with aging, it could itself be a cause of aging, and this process might be reversed by either increasing mitochondrial NAD+ content (with dietary restriction or exercise) or stimulating expression of Sirt3 (for example by inhibiting angiotensin II type I receptors) [6]. However, judging from the fact that the effect of Sirt3 knockdown upon mPTP opening is more prominent in aging hearts than in young hearts [2], Sirt3 may actually be activated as a compensatory mechanism in aging hearts, while aging is mediated by other mechanisms. If so, it remains to be tested to what extent supplementation of NAD+ and Sirt3 would achieve additional protection over the effect of endogenous Sirt3.

Transgenic expression of catalase in mitochondria extends lifespan, retards cardiac aging, and confers stress resistance to the heart [7]. We have shown that genetic deletion of adenylyl cyclase type 5, a cyclic AMP producing enzyme expressed in the brain and the heart, induces both lifespan extension and stress resistance in the heart [8]. The study by Hafner showed that Sirt3, implicated in lifespan extension in humans [9], also inhibits aging and makes the heart resistant to oxidative stress and heart failure [2]. The hormesis hypothesis proposes the idea that the mechanisms of lifespan extension and accumulation of stress resistance are connected [10]. Thus, enhancing a known mechanism of lifespan extension may allow us to retard aging and to achieve stress resistance in the heart. If this is the case, it would be interesting to investigate whether the aforementioned mechanisms of lifespan extension converge at a common mechanism, such as modulation of mPTP, to mediate anti-aging and stress resistance in the heart.
